# (*S*)-*N*-Phenyl-*tert*-butane­sulfinamide

**DOI:** 10.1107/S1600536812020673

**Published:** 2012-05-16

**Authors:** Xiaofei Sun, Xiaoping Zhang, Binbin Zhang, Wenguo Wang, Qingle Zeng

**Affiliations:** a Institute of Green Catalysis and Synthesis, College of Materials and Chemistry & Chemical Engineering, Chengdu University of Technology, Chengdu 610059, People’s Republic of China; b Fujian Institute of Research on the Structure of Matter, Chinese Academy of Sciences, Fuzhou 350002, People’s Republic of China

## Abstract

The asymmetric unit of the title compound, C_10_H_15_NOS, contains two independent mol­ecules with similar conformations. In the crystal, mol­ecules are linked in a head-to-tail fashion by N—H⋯O hydrogen bonds into chains running along the *b* axis. The absolute configuration was assigned on the basis of known chirality of the parent compound.

## Related literature

For the structures of related *N*-alkyl and *N*-aryl alkanesulf­in­amides, see: Datta *et al.* (2008 ▶, 2009*a* ▶,*b* ▶, 2010 ▶); Sun *et al.* (2012*a* ▶,*b* ▶) Zhang *et al.* (2012 ▶); Sato *et al.* (1975 ▶); Schuckmann *et al.* (1978 ▶); Ferreira *et al.* (2005 ▶).
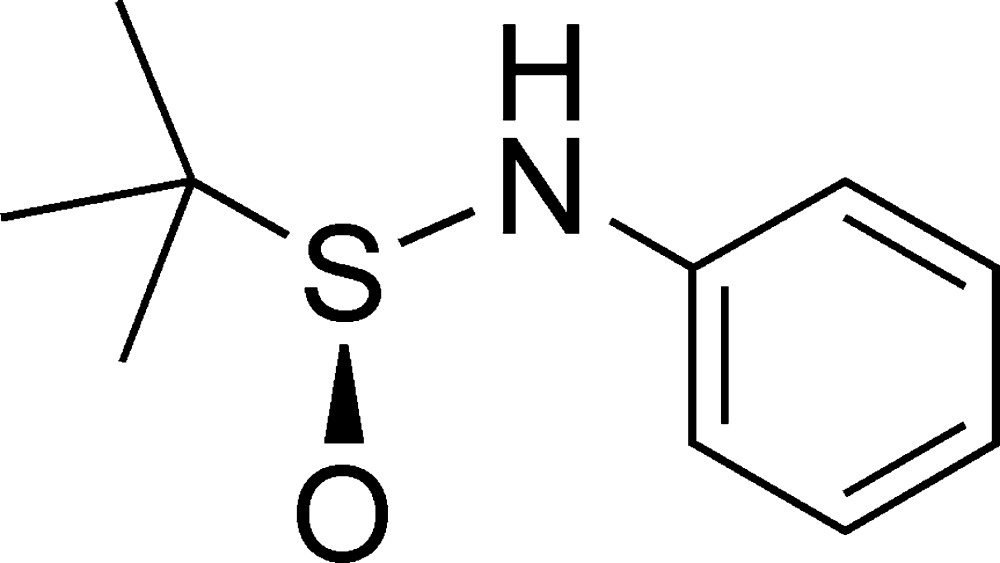


## Experimental

### 

#### Crystal data

C_10_H_15_NOS
*M* *_r_* = 197.29
Orthorhombic, 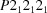

*a* = 9.3596 (4) Å
*b* = 10.4702 (4) Å
*c* = 22.7438 (10) Å
*V* = 2228.82 (17) Å^3^
*Z* = 8
Mo *K*α radiation
μ = 0.25 mm^−1^
*T* = 293 K
0.38 × 0.32 × 0.30 mm

#### Data collection

Aglenet Xcalibur Eos diffractometer
Absorption correction: multi-scan (*CrysAlis PRO*; Agilent, 2011 ▶) *T*_min_ = 0.987, *T*_max_ = 1.000
6065 measured reflections
3993 independent reflections
2503 reflections with *I* > 2σ(*I*)
*R*_int_ = 0.025

#### Refinement

*R*[*F* ^2^ > 2σ(*F* ^2^)] = 0.054
*wR*(*F* ^2^) = 0.090
*S* = 0.98
3993 reflections
249 parameters
H atoms treated by a mixture of independent and constrained refinement
Δρ_max_ = 0.26 e Å^−3^
Δρ_min_ = −0.26 e Å^−3^
Absolute structure: Flack (1983 ▶), 1390 Friedel pairs
Flack parameter: −0.09 (9)

### 

Data collection: *CrysAlis PRO* (Agilent, 2011 ▶); cell refinement: *CrysAlis PRO*; data reduction: *CrysAlis PRO*; program(s) used to solve structure: *SHELXS97* (Sheldrick, 2008 ▶); program(s) used to refine structure: *SHELXL97* (Sheldrick, 2008 ▶); molecular graphics: *OLEX2* (Dolomanov *et al.*, 2009 ▶); software used to prepare material for publication: *OLEX2*.

## Supplementary Material

Crystal structure: contains datablock(s) I, global. DOI: 10.1107/S1600536812020673/rz2751sup1.cif

Structure factors: contains datablock(s) I. DOI: 10.1107/S1600536812020673/rz2751Isup2.hkl

Supplementary material file. DOI: 10.1107/S1600536812020673/rz2751Isup3.cml

Additional supplementary materials:  crystallographic information; 3D view; checkCIF report

## Figures and Tables

**Table 1:** Hydrogen-bond geometry (Å, °)

*D*—H⋯*A*	*D*—H	H⋯*A*	*D*⋯*A*	*D*—H⋯*A*
N1—H1⋯O2^i^	0.80 (3)	2.17 (3)	2.937 (4)	161 (3)
N2—H2⋯O1^ii^	0.83 (2)	2.11 (2)	2.914 (4)	166 (3)

Symmetry codes: (i) 
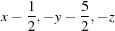
; (ii) 
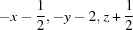
.

**Fig. 1.**
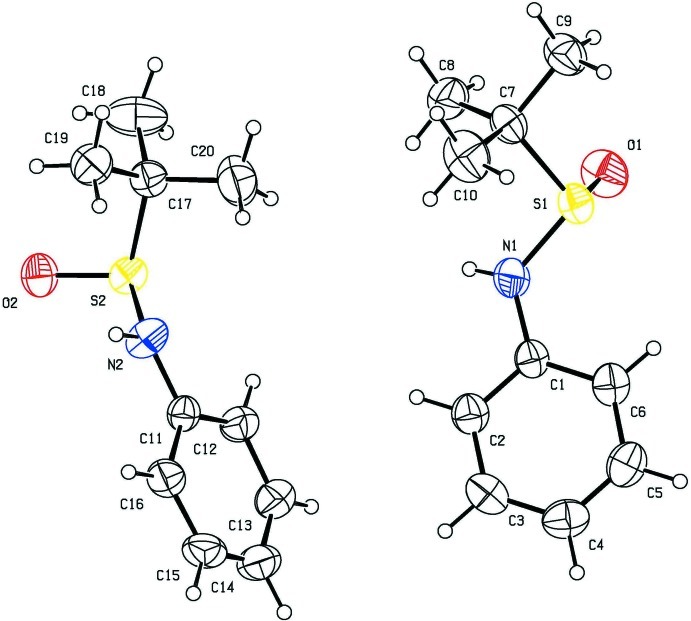
The molecular structure of the title compound, with 50% probability displacement ellipsoids for non-H atoms.

**Fig. 2.**
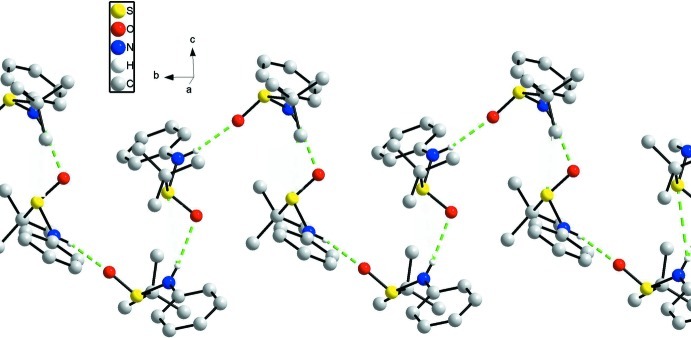
The one-dimensional structure of the title compound. Intermolecular hydrogen bonds are shown as dashed lines.

## supplementary crystallographic information

### Comment

In recent years, sulfonamide moieties have played an increasingly important role in organic chemistry, particularly due to their use as chiral auxiliaries or precursors for the synthesis of a broad family of pharmaceutical agents. As a contribution to this research field, the X-ray crystallographic study of the title compound (Fig. 1) is reported herein. A number of related (*R*)—*N*-(3-methoxyphenyl) *tert*-butanesulfinamides, (*R*)—*N*-(4-biphenyl) *tert*-butanesulfinamide, *N*-aryl alkanesulfinamides and *N*-alkyl alkanesulfinamides have been reported recently (Datta *et al.*, 2008, 2009*a*, 2009*b*, 2010; Sun *et al.*, 2012*a*, 2012*b* Zhang *et al.*, 2012; Sato *et al.*, 1975; Schuckmann *et al.*, 1978; Ferreira *et al.*, 2005).

In the title compound, the value of the *N*-C(aryl) bond (N1—C1 = 1.403 (4) Å; N2—C11 = 1.403 (4) Å) is considerably shorter than those typically found in *N*-alkylsulfinamides (1.470–1.530 Å; Sato *et al.*, 1975; Schuckmann *et al.*, 1978; Ferreira *et al.*, 2005), suggesting a significant delocalization of electrons over the nitrogen atom and the benzene ring. In the crystal structure, the molecules are linked into chains parallel to the *b* axis by intermolecular N—H···O hydrogen bonds (Fig. 2; Table 1).

### Experimental

A oven-dried ground test tube, which was equipped with a magnetic stir bar and fitted with a rubber septum, was charged with (*S*)-*tert*-butanesulfinamide (0.121 g, 1.0 mmol), Pd*_2_*(dba)*3* (0.018 g, 0.02 mmol), *t*Bu-XPhos (0.0212 g, 0.05 mmol) and NaOH (0.08 g, 2 mmol). The vessel was evacuated and backfilled with argon (this process was repeated a total of 3 times) and then phenyl bromide (1.3 mmol), toluene (10 ml) and degassed water (0.3 ml) were added *via* syringe. The solution was stirred at 90° for 20 h. The reaction mixture was then cooled to room temperature, quenched by water, and extracted with ethyl acetate (2 × 20 ml). The organic layer was combined, dried over anhydrous sodium sulfate and filtrated. The filterate was condensed under vacuum. The residual was purified with silica gel column chromatography with a solution of petroleum ether/ethyl acetate (5:1 *v*/*v*) as an eluent to give a white solid (0.167 g, yield 86%). A test tube containing the eluate (petroleum ether/ethyl acetate (5:1 *v*/*v*) was covered with a piece of filter paper and placed motionless at room temperature (about 20°), until a single-crystal was cultured in the bottom of the test tube. M.p.: 383–386 K. [α]D^21^ = +179 (c 3/4, ethyl acetate). Spectroscopic analysis: ^1^H NMR (300 MHz, CDCl_3_), δ (p.p.m.): 7.25–7.24 (m, 2H), 7.01 (t, J = 6.3 Hz, 3H), 5.41 (d, J = 11.1 Hz, 1H), 1.33 (s, 9H). ^13^C NMR (75 MHz, CDCl_3_), δ (p.p.m.): 142.1, 129.1, 122.4, 117.9, 56.3, 22.3. IR (KBr), ν (cm^-1^): 3452, 3145, 2961, 2889, 1598, 1495, 1412, 1363, 1287, 1236, 1053, 887, 751. ESI-MS (negative mode), m/*z* = 196 [M—H]^-^.

### Refinement

The N-bound H atoms were located in a difference Fourier map and refined freely. All other H atoms were positioned geometrically and refined using a riding model, with C—H = 0.93-0.96 Å, and with *U*_iso_(H) = 1.2*U*_eq_(C) or *U*_iso_(H) = 1.5*U*_eq_(C) for methyl H atoms. The absolute configuration was assigned on the basis of known chirality of the parent (*S*)-*tert*-butanesulfinamide compound.

### Crystal data

**Table:**

C_10_H_15_NOS	*D*_x_ = 1.176 Mg m^−^^3^
*M**_r_* = 197.29	Melting point = 383–386 K
Orthorhombic, *P*2_1_2_1_2_1_	Mo *K*α radiation, λ = 0.7107 Å
*a* = 9.3596 (4) Å	Cell parameters from 1915 reflections
*b* = 10.4702 (4) Å	θ = 3.1–29.1°
*c* = 22.7438 (10) Å	µ = 0.25 mm^−^^1^
*V* = 2228.82 (17) Å^3^	*T* = 293 K
*Z* = 8	Block, colourless
*F*(000) = 848	0.38 × 0.32 × 0.30 mm

### Data collection

**Table:**

Aglenet Xcalibur Eos diffractometer	3993 independent reflections
Radiation source: Enhance (Mo) X-ray Source	2503 reflections with *I* > 2σ(*I*)
Graphite monochromator	*R*_int_ = 0.025
Detector resolution: 16.0874 pixels mm^-1^	θ_max_ = 26.4°, θ_min_ = 3.1°
ω scans	*h* = −11→8
Absorption correction: multi-scan (*CrysAlis PRO*; Agilent, 2011)	*k* = −13→10
*T*_min_ = 0.987, *T*_max_ = 1.000	*l* = −16→28
6065 measured reflections	

### Refinement

**Table:**

Refinement on *F*^2^	Secondary atom site location: difference Fourier map
Least-squares matrix: full	Hydrogen site location: inferred from neighbouring sites
*R*[*F*^2^ > 2σ(*F*^2^)] = 0.054	H atoms treated by a mixture of independent and constrained refinement
*wR*(*F*^2^) = 0.090	*w* = 1/[σ^2^(*F*_o_^2^) + (0.0271*P*)^2^] where *P* = (*F*_o_^2^ + 2*F*_c_^2^)/3
*S* = 0.98	(Δ/σ)_max_ = 0.001
3993 reflections	Δρ_max_ = 0.26 e Å^−^^3^
249 parameters	Δρ_min_ = −0.26 e Å^−^^3^
0 restraints	Absolute structure: Flack (1983), 1390 Friedel pairs
Primary atom site location: structure-invariant direct methods	Flack parameter: −0.09 (9)

### Special details

**Table:**

Geometry. All e.s.d.'s (except the e.s.d. in the dihedral angle between two l.s. planes) are estimated using the full covariance matrix. The cell e.s.d.'s are taken into account individually in the estimation of e.s.d.'s in distances, angles and torsion angles; correlations between e.s.d.'s in cell parameters are only used when they are defined by crystal symmetry. An approximate (isotropic) treatment of cell e.s.d.'s is used for estimating e.s.d.'s involving l.s. planes.
Refinement. Refinement of *F*^2^ against ALL reflections. The weighted *R*-factor *wR* and goodness of fit *S* are based on *F*^2^, conventional *R*-factors *R* are based on *F*, with *F* set to zero for negative *F*^2^. The threshold expression of *F*^2^ > σ(*F*^2^) is used only for calculating *R*-factors(gt) *etc*. and is not relevant to the choice of reflections for refinement. *R*-factors based on *F*^2^ are statistically about twice as large as those based on *F*, and *R*- factors based on ALL data will be even larger.

### Fractional atomic coordinates and isotropic or equivalent isotropic displacement parameters (Å^2^)

**Table:**

	*x*	*y*	*z*	*U*_iso_*/*U*_eq_	
S1	−0.63522 (11)	−0.88887 (8)	−0.08184 (4)	0.0658 (3)	
S2	0.04812 (9)	−1.49605 (8)	0.25283 (4)	0.0596 (2)	
O1	−0.6196 (3)	−0.7833 (2)	−0.12453 (11)	0.0943 (9)	
O2	0.0446 (3)	−1.3883 (2)	0.20975 (9)	0.0730 (7)	
N1	−0.5152 (4)	−0.9991 (3)	−0.09407 (14)	0.0781 (10)	
H1	−0.517 (3)	−1.037 (2)	−0.1245 (12)	0.051 (11)*	
N2	0.0029 (4)	−1.4435 (3)	0.31882 (13)	0.0702 (10)	
H2	0.042 (3)	−1.376 (2)	0.3290 (11)	0.038 (9)*	
C1	−0.3963 (4)	−1.0169 (3)	−0.05740 (14)	0.0567 (9)	
C2	−0.3226 (4)	−1.1295 (3)	−0.06151 (15)	0.0697 (11)	
H2A	−0.3507	−1.1910	−0.0887	0.084*	
C3	−0.2065 (5)	−1.1521 (4)	−0.02543 (17)	0.0783 (12)	
H3	−0.1560	−1.2283	−0.0289	0.094*	
C4	−0.1653 (5)	−1.0632 (5)	0.01535 (16)	0.0814 (13)	
H4	−0.0881	−1.0792	0.0400	0.098*	
C5	−0.2383 (5)	−0.9517 (4)	0.01931 (17)	0.0805 (12)	
H5	−0.2106	−0.8912	0.0470	0.097*	
C6	−0.3530 (5)	−0.9262 (3)	−0.01707 (15)	0.0683 (11)	
H6	−0.4007	−0.8485	−0.0144	0.082*	
C7	−0.7985 (4)	−0.9729 (3)	−0.10372 (14)	0.0651 (10)	
C8	−0.7966 (4)	−0.9995 (4)	−0.17018 (14)	0.0892 (12)	
H8A	−0.7820	−0.9209	−0.1911	0.134*	
H8B	−0.8861	−1.0365	−0.1818	0.134*	
H8C	−0.7204	−1.0577	−0.1792	0.134*	
C9	−0.9184 (4)	−0.8808 (3)	−0.08820 (16)	0.0868 (12)	
H9A	−0.9160	−0.8631	−0.0468	0.130*	
H9B	−1.0086	−0.9185	−0.0982	0.130*	
H9C	−0.9061	−0.8027	−0.1097	0.130*	
C10	−0.8088 (4)	−1.0947 (3)	−0.06758 (17)	0.1018 (15)	
H10A	−0.7340	−1.1523	−0.0790	0.153*	
H10B	−0.8999	−1.1343	−0.0743	0.153*	
H10C	−0.7991	−1.0744	−0.0266	0.153*	
C11	−0.1317 (4)	−1.4709 (3)	0.34283 (13)	0.0549 (9)	
C12	−0.2013 (4)	−1.5837 (3)	0.33049 (14)	0.0687 (11)	
H12	−0.1604	−1.6432	0.3052	0.082*	
C13	−0.3322 (4)	−1.6075 (4)	0.35607 (17)	0.0791 (11)	
H13	−0.3804	−1.6823	0.3466	0.095*	
C14	−0.3932 (4)	−1.5244 (5)	0.39494 (17)	0.0868 (13)	
H14	−0.4807	−1.5427	0.4123	0.104*	
C15	−0.3220 (4)	−1.4137 (4)	0.40762 (16)	0.0815 (13)	
H15	−0.3619	−1.3566	0.4343	0.098*	
C16	−0.1937 (4)	−1.3846 (3)	0.38212 (14)	0.0705 (10)	
H16	−0.1481	−1.3080	0.3909	0.085*	
C17	0.2373 (4)	−1.5304 (3)	0.26576 (14)	0.0632 (10)	
C18	0.2876 (5)	−1.5848 (4)	0.20743 (18)	0.1162 (16)	
H18A	0.2232	−1.6508	0.1950	0.174*	
H18B	0.3817	−1.6199	0.2120	0.174*	
H18C	0.2898	−1.5182	0.1785	0.174*	
C19	0.3190 (4)	−1.4111 (3)	0.28162 (15)	0.0825 (12)	
H19A	0.3082	−1.3490	0.2509	0.124*	
H19B	0.4184	−1.4314	0.2863	0.124*	
H19C	0.2825	−1.3768	0.3178	0.124*	
C20	0.2443 (5)	−1.6306 (4)	0.31436 (17)	0.1057 (15)	
H20A	0.2186	−1.5922	0.3512	0.159*	
H20B	0.3397	−1.6639	0.3169	0.159*	
H20C	0.1791	−1.6988	0.3056	0.159*	

### Atomic displacement parameters (Å^2^)

**Table:**

	*U*^11^	*U*^22^	*U*^33^	*U*^12^	*U*^13^	*U*^23^
S1	0.0774 (6)	0.0593 (5)	0.0608 (5)	0.0046 (6)	0.0068 (6)	−0.0011 (5)
S2	0.0663 (5)	0.0637 (5)	0.0488 (4)	−0.0082 (6)	0.0024 (5)	−0.0111 (5)
O1	0.102 (2)	0.0703 (15)	0.1102 (19)	−0.0104 (17)	0.011 (2)	0.0274 (15)
O2	0.0870 (17)	0.0736 (15)	0.0582 (14)	0.0001 (18)	−0.0085 (15)	0.0076 (12)
N1	0.087 (2)	0.092 (2)	0.0550 (19)	0.022 (2)	−0.005 (2)	−0.027 (2)
N2	0.078 (2)	0.071 (2)	0.0618 (19)	−0.024 (2)	0.0131 (19)	−0.0221 (17)
C1	0.067 (2)	0.060 (2)	0.0430 (18)	0.001 (2)	0.0080 (18)	−0.0012 (18)
C2	0.078 (3)	0.067 (3)	0.063 (2)	0.000 (3)	0.003 (2)	−0.0006 (19)
C3	0.082 (3)	0.075 (3)	0.078 (3)	0.017 (3)	0.013 (3)	0.010 (2)
C4	0.077 (3)	0.106 (3)	0.061 (3)	0.002 (3)	−0.003 (3)	0.012 (3)
C5	0.094 (3)	0.088 (3)	0.059 (3)	−0.011 (3)	0.002 (3)	−0.004 (2)
C6	0.087 (3)	0.066 (2)	0.052 (2)	−0.002 (3)	0.007 (2)	0.0038 (19)
C7	0.072 (3)	0.054 (2)	0.069 (2)	0.004 (2)	0.009 (2)	0.0059 (18)
C8	0.087 (3)	0.102 (3)	0.078 (3)	0.004 (3)	−0.009 (3)	−0.017 (2)
C9	0.076 (3)	0.083 (3)	0.101 (3)	0.011 (3)	0.014 (3)	−0.004 (2)
C10	0.116 (4)	0.073 (3)	0.117 (3)	−0.010 (3)	0.024 (3)	0.021 (2)
C11	0.062 (2)	0.057 (2)	0.0462 (19)	−0.001 (2)	0.000 (2)	0.0024 (17)
C12	0.068 (3)	0.073 (3)	0.065 (2)	0.001 (2)	0.012 (2)	−0.0014 (19)
C13	0.071 (3)	0.082 (3)	0.084 (3)	−0.009 (3)	0.007 (3)	0.004 (2)
C14	0.065 (3)	0.117 (3)	0.079 (3)	0.010 (3)	0.014 (2)	0.017 (3)
C15	0.081 (3)	0.097 (3)	0.066 (3)	0.027 (3)	0.017 (3)	−0.004 (2)
C16	0.086 (3)	0.070 (2)	0.055 (2)	0.012 (3)	0.008 (2)	−0.004 (2)
C17	0.068 (2)	0.063 (2)	0.058 (2)	0.002 (2)	0.001 (2)	−0.0055 (18)
C18	0.091 (3)	0.150 (4)	0.107 (3)	0.016 (3)	0.013 (3)	−0.052 (3)
C19	0.071 (3)	0.078 (3)	0.098 (3)	−0.009 (2)	−0.004 (2)	−0.002 (2)
C20	0.105 (3)	0.084 (3)	0.128 (4)	0.000 (3)	−0.019 (3)	0.025 (3)

### Geometric parameters (Å, º)

**Table:**

S1—O1	1.479 (2)	C9—H9B	0.9600
S1—N1	1.634 (3)	C9—H9C	0.9600
S1—C7	1.832 (4)	C10—H10A	0.9600
S2—O2	1.494 (2)	C10—H10B	0.9600
S2—N2	1.654 (3)	C10—H10C	0.9600
S2—C17	1.831 (4)	C11—C12	1.378 (4)
N1—H1	0.80 (3)	C11—C16	1.397 (4)
N1—C1	1.403 (4)	C12—H12	0.9300
N2—H2	0.83 (2)	C12—C13	1.379 (5)
N2—C11	1.403 (4)	C13—H13	0.9300
C1—C2	1.370 (4)	C13—C14	1.365 (5)
C1—C6	1.381 (4)	C14—H14	0.9300
C2—H2A	0.9300	C14—C15	1.368 (5)
C2—C3	1.382 (5)	C15—H15	0.9300
C3—H3	0.9300	C15—C16	1.368 (5)
C3—C4	1.370 (5)	C16—H16	0.9300
C4—H4	0.9300	C17—C18	1.519 (4)
C4—C5	1.356 (5)	C17—C19	1.509 (4)
C5—H5	0.9300	C17—C20	1.526 (4)
C5—C6	1.381 (5)	C18—H18A	0.9600
C6—H6	0.9300	C18—H18B	0.9600
C7—C8	1.537 (4)	C18—H18C	0.9600
C7—C9	1.521 (4)	C19—H19A	0.9600
C7—C10	1.520 (4)	C19—H19B	0.9600
C8—H8A	0.9600	C19—H19C	0.9600
C8—H8B	0.9600	C20—H20A	0.9600
C8—H8C	0.9600	C20—H20B	0.9600
C9—H9A	0.9600	C20—H20C	0.9600
			
O1—S1—N1	110.38 (17)	C7—C10—H10A	109.5
O1—S1—C7	105.27 (16)	C7—C10—H10B	109.5
N1—S1—C7	100.83 (16)	C7—C10—H10C	109.5
O2—S2—N2	109.77 (14)	H10A—C10—H10B	109.5
O2—S2—C17	105.97 (15)	H10A—C10—H10C	109.5
N2—S2—C17	99.62 (16)	H10B—C10—H10C	109.5
S1—N1—H1	119 (2)	C12—C11—N2	121.4 (3)
C1—N1—S1	122.5 (3)	C12—C11—C16	119.3 (4)
C1—N1—H1	118 (2)	C16—C11—N2	119.3 (3)
S2—N2—H2	115.2 (19)	C11—C12—H12	120.4
C11—N2—S2	121.0 (3)	C11—C12—C13	119.2 (4)
C11—N2—H2	117.5 (19)	C13—C12—H12	120.4
C2—C1—N1	118.3 (3)	C12—C13—H13	119.0
C2—C1—C6	119.3 (3)	C14—C13—C12	122.0 (4)
C6—C1—N1	122.5 (4)	C14—C13—H13	119.0
C1—C2—H2A	119.9	C13—C14—H14	120.9
C1—C2—C3	120.2 (4)	C13—C14—C15	118.2 (4)
C3—C2—H2A	119.9	C15—C14—H14	120.9
C2—C3—H3	119.8	C14—C15—H15	119.1
C4—C3—C2	120.5 (4)	C14—C15—C16	121.8 (4)
C4—C3—H3	119.8	C16—C15—H15	119.1
C3—C4—H4	120.4	C11—C16—H16	120.3
C5—C4—C3	119.2 (4)	C15—C16—C11	119.4 (4)
C5—C4—H4	120.4	C15—C16—H16	120.3
C4—C5—H5	119.4	C18—C17—S2	103.5 (2)
C4—C5—C6	121.2 (4)	C18—C17—C20	111.2 (3)
C6—C5—H5	119.4	C19—C17—S2	111.5 (2)
C1—C6—C5	119.6 (4)	C19—C17—C18	111.3 (3)
C1—C6—H6	120.2	C19—C17—C20	112.0 (3)
C5—C6—H6	120.2	C20—C17—S2	107.0 (3)
C8—C7—S1	110.1 (3)	C17—C18—H18A	109.5
C9—C7—S1	104.3 (2)	C17—C18—H18B	109.5
C9—C7—C8	110.6 (3)	C17—C18—H18C	109.5
C10—C7—S1	108.0 (3)	H18A—C18—H18B	109.5
C10—C7—C8	112.4 (3)	H18A—C18—H18C	109.5
C10—C7—C9	111.1 (3)	H18B—C18—H18C	109.5
C7—C8—H8A	109.5	C17—C19—H19A	109.5
C7—C8—H8B	109.5	C17—C19—H19B	109.5
C7—C8—H8C	109.5	C17—C19—H19C	109.5
H8A—C8—H8B	109.5	H19A—C19—H19B	109.5
H8A—C8—H8C	109.5	H19A—C19—H19C	109.5
H8B—C8—H8C	109.5	H19B—C19—H19C	109.5
C7—C9—H9A	109.5	C17—C20—H20A	109.5
C7—C9—H9B	109.5	C17—C20—H20B	109.5
C7—C9—H9C	109.5	C17—C20—H20C	109.5
H9A—C9—H9B	109.5	H20A—C20—H20B	109.5
H9A—C9—H9C	109.5	H20A—C20—H20C	109.5
H9B—C9—H9C	109.5	H20B—C20—H20C	109.5

### Hydrogen-bond geometry (Å, º)

**Table:**

*D*—H···*A*	*D*—H	H···*A*	*D*···*A*	*D*—H···*A*
N1—H1···O2^i^	0.80 (3)	2.17 (3)	2.937 (4)	161 (3)
N2—H2···O1^ii^	0.83 (2)	2.11 (2)	2.914 (4)	166 (3)

Symmetry codes: (i) *x*−1/2, −*y*−5/2, −*z*; (ii) −*x*−1/2, −*y*−2, *z*+1/2.
